# Efficacy of sunscreen with photolyase or regular sunscreen associated with topical antioxidants in treating advanced photodamage and cutaneous field cancerization: a randomized clinical trial^⋆^^⋆⋆^

**DOI:** 10.1016/j.abd.2021.06.005

**Published:** 2022-01-14

**Authors:** Bruno Augusto Alvares, Anna Carolina Miola, Juliano Vilaverde Schimitt, Helio Amante Miot, Luciana Patricia Fernandes Abbade

**Affiliations:** Department of Infectious Diseases, Dermatology, Imaging Diagnosis and Radiotherapy, Universidade Estadual Paulista, Faculty of Medicine, Botucatu, SP, Brazil

**Keywords:** Actinic keratosis, Antioxidants, Deoxyribodipyrimidine photo-lyase, Skin aging, Skin neoplasms, Sunscreening agents

## Abstract

**Background:**

Several treatments are available for skin with advanced photodamage, which is characterized by the presence of actinic keratoses (AK).

**Objectives:**

Evaluate the efficacy of using sunscreen with photolyase compared to regular sunscreen, as well as to compare the combination of a topical formulation of antioxidants versus placebo in the treatment of advanced photodamage.

**Methods:**

This was a randomized, double-blind, factorial clinical trial. Participants with AKs on their forearms were randomized to apply regular sunscreen (SC) or sunscreen with photolyase (SC+P) on both forearms during the day. One of the forearms in each group was randomized again to receive topical antioxidants (AOx), and the other forearm received a placebo cream (both for night application). The four groups were SC/AOx, SC/placebo, SC+P/AOx, and SC+P/placebo. The duration of treatment was 8 weeks. Primary outcomes were total AK clearance, decrease in Forearm Photoaging Scale (FPS), and AK severity scores. Secondary outcomes were reduction in AK count, partial clearance rate, and safety.

**Results:**

Forty participants (80 forearms) were included. All groups showed significant improvement in outcomes at week eight. There were no significant differences between SC and SC+P for either outcome. AOx led to a significant reduction in AK count (22%; p < 0.05). Partial clearance was obtained in 18 (47.4%) forearms treated with AOx and in 9 (23.7%) treated with placebo (p < 0.05). All groups reduced the FPS score, without significant differences among them.

**Conclusions:**

There is no difference in the treatment of advanced photodamage skin when comparing the use of sunscreen with photolyase and regular sunscreen, and topical antioxidants were more efficient in reducing AK count than placebo.

**Study limitations:**

Short interval of follow-up and absence of re-evaluation in the absence of treatment were limitations of the present study.

## Introduction

Skin exposure to ultraviolet radiation (UVR) promotes photoaging and is the main risk factor for the development of actinic keratoses (AK).^1^, ^2^ The UVA spectrum promotes the formation of reactive oxygen species (ROS), leading to oxidative stress and damage to keratinocyte DNA.^2^, ^3^, ^4^ The UVB spectrum is responsible for the formation of pyrimidine dimers in the keratinocyte DNA, the main ones being cyclobutane pyrimidine (CBP) and pyrimidine-pyrimidone photoproducts (6-4PP).^3^

AK diagnosis and treatment is important because it presents a risk of progression to squamous cell carcinoma (SCC).^5^, ^6^ Generally, multiple AKs occur in the same area of photodamaged skin, which accumulates changes in the keratinocyte genetic material, which defines cutaneous field cancerisation (CFC).^7^

Recently, photolyase, a monomeric enzyme not found in the cells of placental mammals, has emerged as an alternative for the treatment of AK and CFC.^8^, ^9^, ^10^ The enzyme has the ability to break down the dimers of CBP and 6-4PP by means of visible light, a process called photoreactivation.^11^ Puig et al. published a review article that concluded that there is evidence to support the beneficial effects in the treatment of AK and CFC with topical photolyase.^2^

Considering the indirect damage caused by UVR, through the formation of ROS, studies with antioxidant agents (AOx) have started. Vitamin C (ascorbic acid) is the most prevalent water-soluble AOx in the epidermis.^12^ It neutralizes free radicals and ROS in the aqueous compartments of the skin and plays an important role in the regeneration of vitamin E (tocopherol).^13^ Vitamin E is a fat-soluble AOx present in the horny layer, produced from the sebaceous glands, characterized by the ability to protect the cell membrane from oxidative stress.^12^ Murray et al. demonstrated that the combination of three AOx (L-ascorbic acid 15%, alpha-tocopherol 1%, and ferulic acid 0.5%), when compared to placebo (vehicle), showed a photoprotective effect by reducing the erythema induced by UVR, significantly reducing the formation of ‘sunburn cells’, as well as reducing the expression of p53 and the formation of thymine dimers.^14^

Most studies using antioxidants formulas focus on its photoprotective properties. Although, no studies have evaluated its efficacy in the treatment of AK. Also, the combination of antioxidants and photolyase has not been described in the literature up to now.

The aim of this study was to evaluate the efficacy of using sunscreen with photolyase compared to regular sunscreen as well as to compare the combination of a topical formulation of antioxidants versus placebo in the treatment of advanced photodamage, characterized by the presence of AK and CFC of the forearms, after an interval of 8 weeks.

## Methods

### Type of study

Randomised Controlled Trial (RCT) with factorial design, open for regular sunscreen and sunscreen containing photolyase and double-blind for topical antioxidant and placebo cream. The study was approved by the institution's ethics committee (nº 2.529.697) and was registered in the Brazilian Registry of Clinical Trials (nº RBR-957zf2). Allocation was made in a 1:1 ratio.

### Participants

Participants of both sexes aged between 60 and 90 years, with a clinical diagnosis of AK in the forearms (three to ten lesions in each forearm) and who had not undergone treatment for AK (except for sunscreen) in the previous six months were included after signing the informed consent form.

The exclusion criteria were as follows: other extensive dermatoses affecting the forearms; hypersensitivity or allergy to any of the substances under study; use of any systemic or topical immunosuppressive substance, oral retinoids, or other local treatments (corticosteroids, anti-inflammatories, retinoids); immunocompromise; and coagulation disorders.

### Interventions

Participants were randomized to receive treatment with sunscreen SPF 99 containing photolyase (SC+P) in multilamellar liposomes and using octocrylene, titanium dioxide, avobenzone, and Tinosorb-S, to be used twice daily with application on both forearms, or regular sunscreen SPF 99 (SC) using as chemical filters Tinosorb-M, Tinosorb-S, avobenzone, octyl-triazone, and 4-methylbenzylidene camphor, with the same SPF of the product containing photolyase, also to be used twice a day on both forearms. These interventions were not performed blindly for participants and researchers, as there was no possibility of hiding the presentations of the products used.

The forearms of the participants in each of the groups mentioned above were randomized to apply moisturizing cream (placebo) to a forearm and cream composed of antioxidant agents formulated with 15% L-ascorbic acid, 1% alpha-tocopherol, and 0.5% ferulic Acid on the Other forearm (AOx), both applied once a day. For these nocturnal interventions, there was blinding for both participants and researchers.

**Figure 1:**
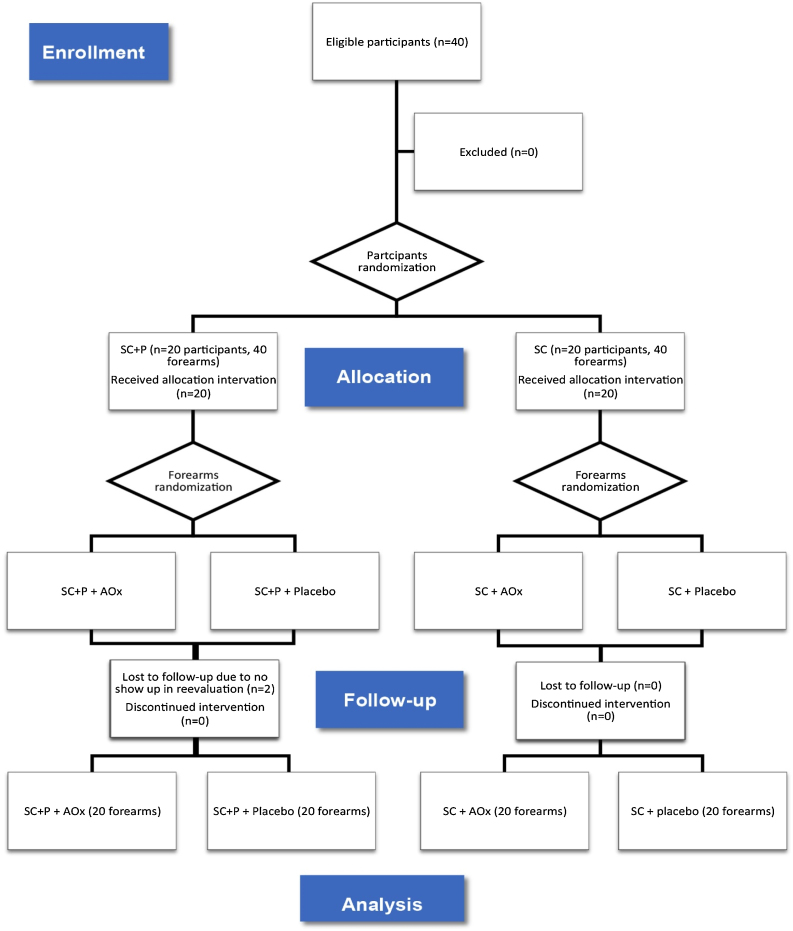
CONSORT flow diagram (ITT evaluation). SC, Regular sunscreen; SC+P, sunscreen containing photolyase; AOx, Topical antioxidant.

Thus, there was the formation of four groups composed of forearms: regular sunscreen and topical antioxidant (SC/AOx), regular sunscreen and placebo (SC/placebo), sunscreen containing photolyase and topical antioxidant (SC+P/AOx), and sunscreen containing photolyase and placebo (SC+P/placebo) (Fig. 1). The placebo and AOx were handled in a three-phase emulsion, white in color, and without odor. Both products were packaged in identical airless packaging, which prevented the oxidation of ascorbic acid contained in one of the formulations. The packages were labeled only with identification topics 1 and 2. After this final randomization, the packages were identified with letters “R” for application of the product on the right forearm and “L” for application on the left forearm so that the patient could identify which side to apply each product. For each patient, the proposed treatments lasted eight weeks. There was a standardization of the areas for counting and evaluating AK lesions and CFC, and this was limited to a line that crosses the metacarpophalangeal joints as a distal limit and a line extending from the antecubital fossa to the lateral epicondyle of the forearms as the proximal limit.

### Outcomes

Primary outcomes were total AK clearance rate, reduction in CFC activity assessed by improving the Forearm Photoaging Scale (FPS)^1^ and forearm AK Severity Score (AKSS)^15^ after 8 weeks of treatment.

Secondary outcomes were reduction in the AK count, partial clearance rate (reduction of 50% or more), adverse effects related to treatment, tolerability, and the appearance of non-melanoma skin tumors.

### Sample calculation

The sample size was calculated to detect a reduction in a difference of more than 10% between the groups and an equivalent standard deviation of the differences. A power of 0.9, alpha of 0.05, and dropout estimate of 10% were adopted, resulting in 40 patients (80 forearms).

### Randomisation and evaluations

Participants and forearms were randomized into blocks using computer simulation. After computational randomization, the combinations of treatments generated were separated individually in sealed envelopes. The envelopes were kept with the researcher, and, at the time of inclusion, an envelope was randomly selected to guide the patient’s treatment.

The participants were evaluated at two-time points: T0, inclusion, randomization, clinical evaluation, and beginning of treatments; T60, clinical evaluation, evaluation of adverse effects, and tolerability.

At enrolment, the AKs were counted and marked with a pen for each forearm, respecting the anatomical limits considered above, the evaluation form was filled out, and the forearm photoaging scale and forearm AK severity score was applied.

The FPS considers the number of superficial and hypertrophic AKs, amount and severity of wrinkles, solar lentiginosis, visible purple, starred atrophic scars, elastosis, and loss of elasticity. All parameters are added together to form a scale ranging from 0 to 192, and higher total values indicate greater photodamage.^1^

AKSS considers the number of lesions and the following characteristics: degree of erythema, hyperkeratosis, infiltration, and diameter (measured in millimeters) of the AKs. Each of the criteria is graded from 0 to 3. The lesions were characterized individually, and the values were added to obtain the total in the studied area. The higher the total value, the greater the severity of the AKs and CFC.^15^ This severity score was published and validated for AK and CFC of the head. Recently, this score was applied to the evaluation of AK of the forearms, and its validation is ongoing.^16^

Evaluations of patients at both times were performed by the same evaluator who was blinded to the treatments of topical antioxidants and placebo. Patients were also unaware of which formula was a placebo and topical antioxidant. At the end of the study, group allocation was revealed.

### Statistical analysis

All participants included in the study and randomized were part of the Intention-To-Treat (ITT) population. Data analysis was performed for the ITT population using a generalized mixed-effects linear model that deals with the missing data in its analytical structure.^17^

Categorical variables were presented as absolute numbers and percentages. Continuous variables were assessed for normality by the Shapiro-Wilk test and represented by means and standard deviations, or medians and quartiles (p25–p75).

The number of AKs, the forearm photoaging scale, and the forearm AK severity score were compared for time and groups (over time) using the generalized linear model of mixed effects, robust covariance structure, covariance matrix autoregressive type 1, *post-hoc* comparison with Sidak comparison, and negative gamma or binomial probability adjustment, when indicated.

The effect size was calculated as the difference in the averages of each variable at T60 and T0, and the confidence interval using the bootstrap technique with 1000 resamples.

Data were analyzed in IBM SPSS 25v. Significance was set at p < 0.05.

## Results

Between January 2019 and January 2020, 40 participants were eligible and randomized for the study. Twenty forearms were part of the SC+P/AOx group and the SC+P/placebo group. Twenty forearms were part of the SC/AOx and SC/placebo groups. There were two dropouts in the SC+P group, not associated with the interventions, as patients did not return for re-evaluation on the scheduled date (Fig. 1).

The main clinical and demographic data of the 40 participants at baseline (T0) are displayed in Table 1. There was no significant difference between groups for any of the variables.

**Table 1:** Main demographic characteristics of the participants in the inclusion (T0) according to the randomized group.

Variable	SC	SC+P	Total	p-value
Sex – n (%)				0.519
Male	9 (45)	7 (35)	16 (40)	
Female	11 (55)	13 (65)	24 (60)	
Phototype – n (%)				0.08
I	- (-)	1 (5)	1 (3)	
II	9 (45)	13 (65)	22 (55)	
III	11 (55)	6 (30)	17 (43)	
Age (years)^a^	73 (7)	73 (9)	73 (8)	0.938
Schooling – n (%)				0.742
Illiterate	2 (10)	2 (10)	4 (10)	
Elementary	15 (75)	16 (80)	31 (78)	
High School	3 (15)	2 (10)	5 (13)	
FPS^b^	105 (93–114)	110 (78–118)	107 (91–116)	0.391
AK Count (T0)^b^	7 (6–9)	7 (6–8)	7 (6–9)	0.321
AKSS (T0)^b^	76 (46–100)	70 (52–91)	72 (51–95)	0.459

SC, Regular sunscreen group; SC+P, Sunscreen group with photolyase; AK, actinic keratosis; FPS, forearms photoageing scale; AKSS, forearms AK severity score.

a Mean (Standard Deviation).

b Median (p25–p75).

There was no significant difference in primary and secondary outcomes between the regular sunscreen and sunscreen containing photolyase groups (Table 2). Total clearance was achieved by three forearms treated with topical antioxidant (7.9%; 95% CI 2.6–13.2%) and one forearm treated with placebo (2.6%; 95% CI 0–7.9%), without a difference (p = 0.997). Partial clearance was achieved by 18 forearms treated with topical antioxidant (47.4%; 95% CI 34.2–60.5%) and nine forearms treated with placebo (23.7%; 95% CI 13.2–34.2%; p = 0.018).

**Table 2:** Main clinical outcomes were resulting from treatments.

	SC		SC+P	
	AOx	Placebo	AOx	Placebo
**T0**				
AK count^c^	7 (6–10)	7 (7–9)	7 (6–9)	6 (6–8)
FPS^c^	105 (94–114)	105 (94–114)	113 (73–117)	108 (78–117)
AKSS^c^	81 (46–95)	69 (46–111)	70 (53–92)	70 (52–89)
**T60**				
Total clearance^d^	2 (10)	1 (5)	1 (5)	– (–)
FPS^c^	79 (66–93)^a^	78 (68–93)^a^	81 (68–94)^a^	85 (74–95)^a^
AKSS^c^	34 (13–51)^a^	38 (18–62)^a^	20 (11–30)^a^	32 (22–43)^a^
AK count^c^	4 (3–6)^a^, ^b^	5 (4–7)^a^	4 (2–5)^a^, ^b^	5 (3–7)
Partial clearance^d^	8 (40)^b^	5 (25)	10 (50)^b^	4 (20)

SC, Regular sunscreen group; SC+P, Sunscreen group with photolyase; AOx, Group with topical antioxidant; FPS, forearm photoageing scale; AKSS, forearm AK severity score.

a p < 0.05 (T0 vs. T60).

b p < 0.05 (AOx vs. Placebo).

c Median (p25-p75).

d n (%).

**Figure 2:**
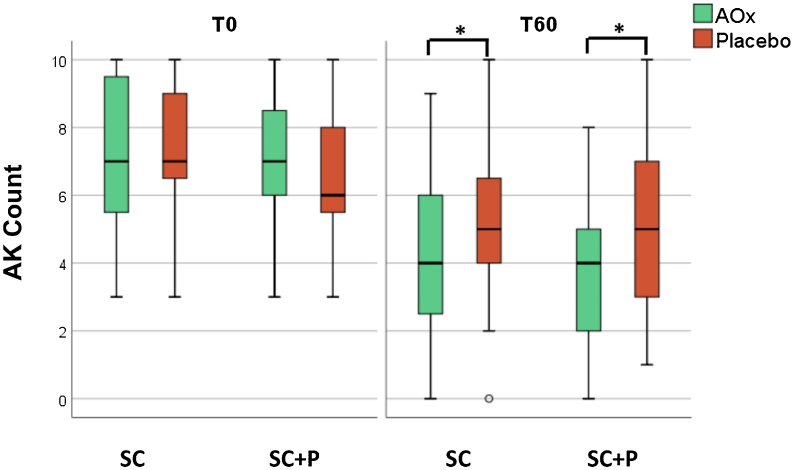
AK count in the four treatments groups at T0 and T60. AK, actinic keratoses; SC, regular sunscreen SPF 99; SC+P, sunscreen containing photolyase SPF 99; AOx, cream composed of antioxidant agents. (*p < 0.05).

There was a reduction in the AK count in all groups over time (p < 0.05). In addition, there was a reduction in the AK count in the groups that used topical antioxidants (SC/AOx and SC+P/AOx) in relation to those who used a placebo (p < 0.05) (Table 2 and Fig. 2).

The FPS decreased for all groups over time (p < 0.05), regardless of the treatment group (Table 2). There was no statistically significant difference between the other interventions for this outcome.

Likewise, there was a reduction in the AKSS for all groups over time (p < 0.05), without superiority for any treatment (Table 2).

The effect sizes for the outcomes are shown in Table 3. The difference in the AKSS means and partial clearance was higher in the groups that used sunscreen with photolyase and topical antioxidant (SC+P/AOx) compared with the groups that used the placebo.

**Table 3:** Percentile difference (T60-T0) in the studied outcomes (means and 95% Confidence Intervals).

	SC		SC+P		Total
	AOx	Placebo	AOx	Placebo	
Total clearance^a^	10 (0–25)	5 (0–15)	6 (0–17)	– (–)	5 (1–11)
FPS^b^	22 (15–29)	20 (14–26)	22 (12–31)	17 (8–26)	20 (17–24)
AKSS^b^	45 (32–59)	32 (21–42)	53 (42–65)^c^	34 (27–41)	41 (35–47)
AK count^b^	2.9 (1.8–4.0)	2.0 (1.2–2.8)	3.6 (2.6–4.6)	1.1 (0.4–1.8)	2.4 (1.9–2.9)
Partial clearance^a^	40 (25–60)	25 (10–40)	56 (39–72)^c^	22 (11–33)	36 (26–45)

SC, Regular sunscreen group; SC+P, Sunscreen group with photolyase; AOx, Group with topical antioxidant; FPS, forearms photoageing scale; AKSS, forearms AK severity score.

a Percentage of clearance.

b Average of the differences.

c Value p < 0.05, SC+P/AOx group compared with placebo.

### Safety and tolerability

The itching was described with the use of a topical antioxidant and a placebo by one participant; another participant described itching with the use of sunscreen containing photolyase. There were no reports of erythema, oedema, ulceration, or blisters, and the proposed treatments were well tolerated. There was a basal cell carcinoma (BCC) on the left forearm of one of the participants where he was applying sunscreen containing photolyase and topical antioxidants. Another participant developed squamous cell carcinoma (SCC) on the left forearm, where he was applying regular sunscreen and topical antioxidants.

## Discussion

The authors of the present study found no difference in the treatment of advanced photodamage and CFC when comparing the use of sunscreen with photolyase and regular sunscreen without photolyase. However, the use of an antioxidant formula gave a reduction in the AK count, which reflects a reduction in CFC activity. Although topical antioxidant is frequently used as an anti-aging product, the present study showed improvement in AK count without a significant change in other aspects of the forearm photoaging scale.

When interventions were analyzed over time, all parameters showed statistically significant improvements. The reduction or resolution of AKs can occur in up to 21% of patients within one year.^18^ In the present study’s clinical trial, all participants used at least regular sunscreen, with a reduction or resolution of the AKs in the forearms of 38.7%. In addition, two articles found that regular use of sunscreen reduced the AK count by up to 25% in immunocompetent patients and 50% in patients with transplanted solid organs.^19^, ^20^

Puig et al. analyzed the evidence available in the previous ten years of using sunscreen containing photolyase. The authors analyzed 11 studies, totaling 228 participants, only three of which were RCTs, and concluded that there was evidence to support the use of photolyase for the treatment of AK and CFC.^2^

One of the clinical trials was that of Moscarela et al., who compared the use of sunscreen with photolyase and regular sunscreen SPF 50 in patients with four or more AKs on the face and scalp. The study included 50 participants but had 14 dropouts, resulting in 17 participants who used sunscreen with photolyase and 19 regular sunscreen SPF 50. After six months of follow-up, there was a reduction in the AK count in both groups, which did not reach statistical significance. When evaluating the subgroup of participants with up to ten AKs (n = 20), the sunscreen group with photolyase proved to be superior in reducing the AK count. In this subgroup, 14% of participants who used sunscreen with photolyase had new lesions versus 54% in those who used regular sunscreen SPF 50.^21^ In the present study, only one patient in the sunscreen with photolyase group presented with new AKs versus none in the regular sunscreen group.

Eibenschutz et al. conducted a clinical trial with 30 participants to evaluate the effectiveness of sunscreen with photolyase versus regular sunscreen in the treatment of AK and CFC of the face and scalp after one session of MAL-PDT. An increase in the number of AKs was observed after nine months of treatment in the participants who used regular sunscreen, while there was a reduction in the AKs in those who used sunscreen with photolyase (p = 0.001).^22^

Puig et al. developed a prospective, controlled, non-randomized study with 13 participants with AKs in exposed areas to evaluate the effectiveness of sunscreen with photolyase versus regular sunscreen for 4 weeks in the treatment of AKs and CFC. The groups were formed by nine participants in the sunscreen with photolyase and three in the regular sunscreen. The study analyzed the following outcomes: clinical improvement, dermatoscopy, immunohistochemistry, and appearance on confocal microscopy. There was a statistically significant improvement in all the outcomes studied in the group that used sunscreen with photolyase, but there was no improvement in the group that used regular sunscreen. However, this was a non-randomized study with a low number of participants, and the AK count was not analyzed as an outcome, in addition to presenting a disproportionate distribution between the groups, which compromises the data validity.^11^

The RCT did not show superior efficacy of sunscreen containing photolyase in the treatment of AKs of the forearms of immunocompetent patients, which contradicts the results of previous studies.

The use of antioxidant substances associated with sunscreen has become frequent since several studies have demonstrated a photoprotective effect by reducing erythema induced by UVR, reducing the expression of p53, reducing the formation of ‘sunburn cells’, and decreasing the expression of immunosuppressive cytokines.^14^

Vitamin C neutralizes free radicals in the aqueous compartments of the skin, in addition to playing an important role in the regeneration of vitamin E.^12^, ^13^ The main function of vitamin E is to protect cell membranes from oxidative stress.^12^

The association of 15% L-ascorbic acid with 1% alpha-tocopherol demonstrated that they have a synergistic effect on the skin. When alpha-tocopherol neutralizes oxidative stress in lipids, its oxidized products can be regenerated by L-ascorbic acid. This interaction helps to renew antioxidant protection in tissues. The formulation containing the two products doubled the UV protection for the skin when compared to L-ascorbic acid alone.^23^, ^24^ Lin et al. demonstrated that the addition of 0.5% ferulic acid increased both the stability of the formula and doubled the UV protection for the skin. It is postulated that ferulic acid protects L-ascorbic acid and alpha-tocopherol, undergoing oxidation before the others in the formula.^25^

There have been no studies evaluating the use of topical antioxidants for the treatment of AK and CFC. The present study demonstrated a beneficial and safe effect of the combination of 15% L-ascorbic acid, 1% alpha-tocopherol, and 0.5% ferulic acid in reducing the number of AKs, consequently controlling CFC activity.

One of the limitations of this study was that it did not directly compare the regular sunscreen and sunscreen with photolyase groups, making this comparison inferior to the topical antioxidant versus placebo comparison. In addition, the study included only patients with AK counts between three and ten, and the results may not apply to those patients with greater numbers of lesions as well as immunosuppressed patients.

The authors acknowledge that the 60-day follow-up interval might be related to the poor results of photolyase. Perhaps to achieve better results, its use should be prolonged. However, further studies must be carried out to confirm this hypothesis. In addition, as demonstrated by Eibenschutz et al.,^22^ it is possible that photolyase has the best indication for preventing the recurrence of AK treated by other methods, being an alternative to stabilize but not treat CFC.

The authors also believe that prolonged use of topical antioxidants might improve other signs of photoaging, since studies showed that reduction in the transcription of matrix metalloproteinase-1 inhibits the formation of thymine dimers, reducing collagen breakdown process and carcinogenesis.^26^

The strength of the present study was the possibility of evaluating the effects of three interventions in a factorial study design in a group of immunocompetent patients who frequently present AK in their forearms.

## Conclusions

Sunscreen (SPF 99) containing photolyase was not superior to regular sunscreen (SPF 99) in terms of the studied outcomes after eight weeks.

The use of the topical antioxidant formula is safe, tolerable, and also had more efficacy in reducing the AK count in the forearms when associated with regular sunscreen or sunscreen with photolyase.

## Financial support

This study received financial support from the Sao Paulo State Dermatology Support Fund (FUNADERSP), no. 73-2018.

## Authors’ contributions

Bruno Augusto Alvares: Conception and design of the study; survey of data, or analysis and interpretation of data; statistical analysis; writing of the article or critical review of important intellectual content; obtaining, analysis and data interpretation; effective participation in research guidance; intellectual participation in propaedeutic and/or therapeutic conduct of studied cases; critical literature review; final approval of the final version of the manuscript.

Anna Carolina Miolla: Writing of the article or critical review of important intellectual content; obtaining, analyzing and interpreting the data; effective participation in research guidance; intellectual participation in propaedeutic and/or therapeutic conduct of studied cases.

Juliano Vilaverde Schimitt: Conception and design of the study; statistical analysis; writing of the article or critical review of important intellectual content; effective participation in research guidance; intellectual participation in propaedeutic and/or therapeutic conduct of studied cases; critical literature review.

Helio Amante Miot: Conception and design of the study; survey of data, or analysis and interpretation of data; statistical analysis; writing of the article or critical review of important intellectual content; obtaining, analysis and data interpretation; effective participation in research guidance; intellectual participation in propaedeutic and/or therapeutic conduct of studied cases; critical literature review; final approval of the final version of the manuscript.

Luciana Patricia Fernandes Abbade: Conception and design of the study; survey of data, or analysis and interpretation of data; statistical analysis; writing of the article or critical review of important intellectual content; obtaining, analysis and data interpretation; effective participation in research guidance; intellectual participation in propaedeutic and/or therapeutic conduct of studied cases; critical literature review; final approval of the final version of the manuscript.

## Conflicts of interest

None declared.
